# Inducing and measuring identity levels in professional rugby players during immersion in an interactive virtual environment

**DOI:** 10.1371/journal.pone.0359964

**Published:** 2026-10-07

**Authors:** Solène Neyret, Julien Pellet, Julien Ryard, Mickaël Campo, Jean-Rémy Chardonnet

**Affiliations:** 1 Arts et Metiers Institute of Technology, Laboratoire d’Ingénierie des Systèmes Physiques Et Numériques, Hautes Ecoles Sorbonne Arts et Métiers Université, Université Bourgogne Europe, Chalon-Sur-Saône, France; 2 Université Bourgogne Europe, Psy-DREPI UR 7458, Centre d’Expertise de la Performance Mentale, Dijon, France; 3 Fédération Française de Rugby, Sport Psychology Department (Pôle PMAS), Marcoussis, France; Gabriele d’Annunzio University of Chieti and Pescara: Universita degli Studi Gabriele d’Annunzio Chieti Pescara, ITALY

## Abstract

During a game, players experience substantial affective fluctuations, particularly through cognitive appraisal processes, which notably involves self-categorisation process. However, designing a mental training programme using the contextual elements of a game is still a challenge. Moreover, social contexts can be recreated in virtual reality and it has been demonstrated that people tend to have realistic affective and behavioural reactions to it. In this study, we recreated, in virtual reality, one of the key contexts that players can encounter during a rugby game, a try. After an embodiment phase, participants were standing on the field with other avatars representing the other players, surrounded by an audience of virtual supporters. We used different visual and auditory parameters creating two different kinds of social feedback. In the first condition the social feedback was aimed at inducing social identity, in the second condition the social feedback received in the virtual environment was aimed at inducing personal identity. We measured participants’ identity level before and after each condition, using interactive visual analogue scales inside the virtual environment. We also measured levels of presence and embodiment using the same method. Affective states were measured using an interactive adaptation of Russel’s affect grid, inside the virtual environment as well. Our experiment presents an innovative method aimed at inducing and measuring different identity levels in virtual reality.

## Introduction

Performance in team sports is a complex phenomenon that depends on various factors, such as group dynamics [1–3] and emotions [4–7]. It has been shown that these factors influence each other. In particular, research in social and sport psychology has shown that group membership can shape affective responses through individuals’ identification with their team [5–8]. Thus, contextual elements influencing social identity salience during a game participate in the players’ emotional experience, which encompasses an arousal dimension, as well as a valence (pleasant) dimension [9–11]. This includes factors associated with enhancing or diminishing perceived social value of individuals [3,12,13], with making collective goals salient [6], or with contextual cues focusing on the presence of the outgroup or the ingroup [14–16]. Therefore, it is essential for coaching staff to be equipped to help athletes manage identity-driven affective processes, in order to support effective regulation and optimise performance [7,17,18].. One innovative tool to achieve this is Virtual Reality (VR). With the appropriate multisensory cues during the immersion, VR offers the possibility to recreate specific environments in which practitioners can manipulate contextual elements to elicit targeted affective responses by activating identity-based mechanisms. The aim of this study is thus to test a VR method to induce and measure affective states in rugby players. Therefore, VR was used as an immersive and controlled experimental setting to examine whether identity-related social cues could influence players’ short-term social identity and affective states.

### Social identity as a mean to induce emotions

Social identity [13] is part of a broader identity process called the Social Identity Approach [3,12], which proposes that individuals, such as team sport players, can perceive themselves through different identity levels, such as individuals (as an “I”) or as group members (as a “We”). These social cognitions influence how they understand and process information coming from their environment. When players perceive themselves as group members, their cognitive framework operates on the principle that what benefits the group is inherently beneficial to themselves. Conversely, when they consider themselves as individuals, personal matters drive their cognition [8]. Thus, the literature on antecedents of social identity mainly refers either to the group’s contribution to perceived social value [12,13], and to a perception of a logically coherent context, notably in terms of expectations in interactions [19]. Recent developments regarding identity processes (i.e., identity complexity; [20,21]) suggest that identity levels may not simply alternate in salience but can coexist, overlap, or even conflict. However, to date, little is known regarding the interplay of the identity levels.

A growing body of literature in social and sport psychology has established the close interdependence between social identity and emotional experience [22,23] especially in sports (see [24] for a review). Individuals tend to experience emotions not only as private, internal states but also as socially embedded responses shaped by their group memberships and the salience of those identities in context. When athletes strongly identify with their team, emotional reactions to success, failure, or interpersonal events are often intensified and more aligned with the group’s values and goals [4,7]. Specifically, previous research highlighted a specific link between higher levels of social identity and pleasant affects have been observed multiple times [4,5,21].

Building on this framework, mobilising identity-based processes has emerged as a promising avenue for deliberately inducing affective states relevant to performance. However, a key challenge lies in the degree to which individuals can be immersed in identity-relevant contexts in a controlled and replicable manner. In this respect, VR offers a powerful tool: it enables the creation of highly customisable environments that can simulate team-based contexts while varying key elements likely to enhance or attenuate feelings of group belonging. This opens up new possibilities for studying, and eventually training, athletes’ emotional responses by systematically activating or deactivating identity cues.

### Virtual reality as a tool to induce social identity

VR offers the possibility to fully create an environment and to control every aspect of it. For example, contexts which exist uniquely during the limited time window of a game, such as a stadium full of supporters, can be recreated in VR and used for training. It also allows to present one situation in different social contexts, for instance a game situation with teammates and supporters wearing the national team’s colours and the same game situation with teammates and supporters wearing neutral colours). Thus, it allows to manipulate diverse factors or sensory cues that can elicit social or personal identity. Nonetheless, in order to obtain these effects, participants need to experience the sensations of presence in the virtual environment (VE) and body ownership illusion over their virtual body.

#### Body ownership illusion.

Body ownership illusion refers to the sensation of owning a body or a “body part” that does not belong to one’s body. It has been shown that this sensation is linked to the properties of multisensory integration in the human brain, especially the integration of tactile and visual information [25]. In VR, it is possible to elicit a body ownership illusion over a virtual body using the characteristics of multisensory integration [26]. If the visual information associated with the virtual body is synchronized with the perceived tactile or motor information associated with the real body, then the body ownership illusion can be elicited. More precisely, in VR, if the movements of the virtual body are synchronized with the movements of the participant, they will experience a sense of body ownership and agency over this virtual body through visuomotor integration [27]. In the field of VR studies, it has been demonstrated that visual sensorimotor contingencies, first-person perspective, spatial collocation, and morphological appearance of the virtual body are the key factors in eliciting body ownership over a virtual body inside a VE [28–30]. In this experiment, we use visuo-motor correlation to trigger the illusion of body ownership towards the virtual body. The movements of the participant’s upper body are tracked and mapped to the virtual body, creating a sense of integration between motor activity and visual feedback received inside the VE.

#### Presence.

Presence is defined as a phenomenal subjective experience of “now being there”. It includes a temporal and spatial self-reference [31]. Two main components of presence in VR have been described, place illusion and plausibility illusion. place illusion is defined as the subjective spatial self-location in the place depicted by the VE, and plausibility illusion is defined as the subjectively experiencing that the events taking place inside the VE are “really happening” [32–34]. The temporal aspect is common to both place illusion and plausibility illusion as both illusions last only while the participant is immersed inside the VE [35].

One important issue of measuring presence and body ownership illusion with questionnaires filled in after immersion lies in the remanence of the participants’ feeling: the (past) moment of immersion is already gone. Therefore, participants can only answer according to the memory they have of the sensation experienced while immersed in VR. The solution is then to measure presence during the immersion of the participants in VR. However, this presents one difficulty, consisting in the observation that exogenous elements, not belonging to the VE, can easily break the sensation of presence.

The term “co-presence” refers to the subjective phenomenal experience of being inside the VE with other virtual avatars. As it shows the same properties of temporality as presence and body ownership illusion, it is interesting to measure it as well during the immersion in VR, and without breaking it.

It thus becomes essential that any VR tool designed to modulate levels of social identity ensures the induction and maintenance of key immersive components—namely, the illusion of body ownership, the sense of spatial presence, and the experience of co-presence within the VE in which athletes are immersed. As measuring identity levels or affects can break the immersion in the VE, these illusions would need to be maintained during the measures steps which are essential to provide evidence of tangible effects of the variables controlled by researchers. Presence is of importance as it has been shown in previous research that participants can feel an “in-group affiliation” with virtual people and that this effect influences their behaviours and emotional states [36].

Accordingly, the aim of this study is to test a VR method to induce and measure affective states in rugby players using the framework of identity processes. We hypothesised that, following immersion in the VE under the collective condition (Coll.), participants would report higher levels of social identity, physiological arousal, and pleasant affect, as well as greater co-presence compared to the individual condition. Conversely, we expected that immersion in the individual condition (Indiv.) would lead to an increase in personal identity salience, accompanied by lower levels of arousal and pleasant affect. Finally, we anticipated that core components of immersive experience—namely, place illusion, plausibility illusion, and body ownership illusion—would remain stable across both conditions.

## Materials and methods

### Participants

Forty male professional rugby players, from three different clubs, voluntarily participated in the experiment (*M*_*age*_ = 21.38; *SD* = 2.94). Recruitment was conducted by direct contact with the responsible of each training centre. All participants had normal vision and were screened for contraindications regarding the use of VR through pre-experimental forms (e.g., epilepsy, recent alcohol intake, psychoactive drug treatment, recent cerebral concussion). Data from five participants were excluded due to technical issues and from one participant due to recent cerebral concussion. In total, data from 35 participants, including 7 professional national players, 6 professional international players, and 24 aspiring professional players (currently training in a national training centre and occasionally competing at the professional level without being part of the main squad) were collected. The study was approved by the French ethic committee of sports studies (CERSTAPS RB00012476-2022-02-05-178). Data were collected from August 22, 2022, to February 15, 2023.

### Equipment

#### Virtual reality equipment.

The Head-Mounted-Display (HMD) used in this experiment was an HTC Vive Pro. It displays a 3D scene in stereo with a field of view of 110 degrees. The device uses two screens, one per eye, each of them having a display resolution of 1440 x 1600 pixels (for each eye), 2880 × 1600 pixels in total. It has a double 3.5-inch 90 Hz AMOLED screen and weighs 500g. A single-use protection was used to protect the inside foam part of the HMD, and was changed after each participant, for sanitary reasons.

For the audio, we used the integrated and adjustable audio system of the HTC Vive pro. The HMD was connected through USB-C 3.0, Display Port 1.2 and Bluetooth to the computer (see hardware specifications below). Three infrared cameras were installed in each room where the experiment was performed (infrared station V2). Five trackers V2 were used. The participant was equipped with one tracker on each hand, and therefore, could interact with the environment by moving his hands naturally, hence no controllers were needed. The other trackers were placed as follows: one on each foot of the participant and one under his belly button.

#### Computer.

The computer we used for connecting the HTC Vive Pro and executing the VR application was an MSI portable notebook, with a processor intel Core^TM^ i7-7700HQ CPU @2.80Gz 2.80Gz, 16 Go RAM, and a NVidia GeForce GTX 1070 graphic card.

### Virtual environments

#### Tools and software.

The VE was programmed and generated under Unity3D (Unity Technologies, www.unity.com). The tracking and calibration of the whole HTC Vive Pro VBR system was done using SteamVR, runtime: version 1.22.13. All the avatars of the application (participant, teammates, opponents, and referee) were generated using the “Make human” software. Make Human (www.makehumancommunity.org) is a free and open-source software for 3D modelling of human avatars, it allows to create avatars compatible with the Unity3D engine. These avatars are created with a standard skeleton linked to its mesh. This makes them compatible with traditional animation tools, from Unity, for playing animations or animating the avatars with inverse kinematics. This software allows creating avatars of different height, weight, gender, age, colour and hairdo. It also allows adding different types of clothes. This allowed us to generate enough avatar models for constituting two rugby teams of fifteen different individuals. For inverse kinematics, we used Final IK. This software library allows the calculation of inverse kinematic actions applied to virtual skeletons. It provides advanced tools for humanoid skeletons, adapted to the participant’s avatar. It is available as a Unity3D package in the Unity assets store.

#### Virtual reality calibration.

With the equipment, the participants’ movements were synchronized in real time with the movements of their virtual body. That way, the motor output of the participants was synchronized with the visual input they perceived when looking at their virtual body. We know from previous studies that seeing one’s virtual body reflected in a virtual mirror in front of oneself inside VR is an important factor in inducing body ownership illusion [37]. Therefore, we decided to include an embodiment phase before the VR scene. In this embodiment phase, we placed a mirror on the virtual rugby field and participants were instructed to move their hands and legs while looking at the mirror in front of them (see S2 Fig). We used several models of virtual rugby players (avatars) to compose the two different rugby teams. The participant’s avatar was created based on one rugby player model; only the skin colour was changed to match the participant’s skin colour. Therefore, the avatar that participants saw in the mirror did not look like them. However, it has been shown that, when participants experience body ownership illusion over a virtual body, they accept this body as the partial representation of their “self”, during the limited time of the experiment, even if the avatar does not look like them [38].

#### Virtual reality scenario.

The VR scene consisted of the reproduction of the Stade de France, using an adaptation of a stadium model bought on the Unity assets store. The stadium features a giant screen with a replay video and written information about the playing teams and the score. The logos and supporters’ colours were adapted to the colours of each team. The behaviour of the virtual teammates, opponents and referee were determined with the help of three coaches from the French Federation of Rugby, and the head of sport psychology department of the French Rugby Federation. Fig 1 displays a view of the stadium.

**Fig 1:**
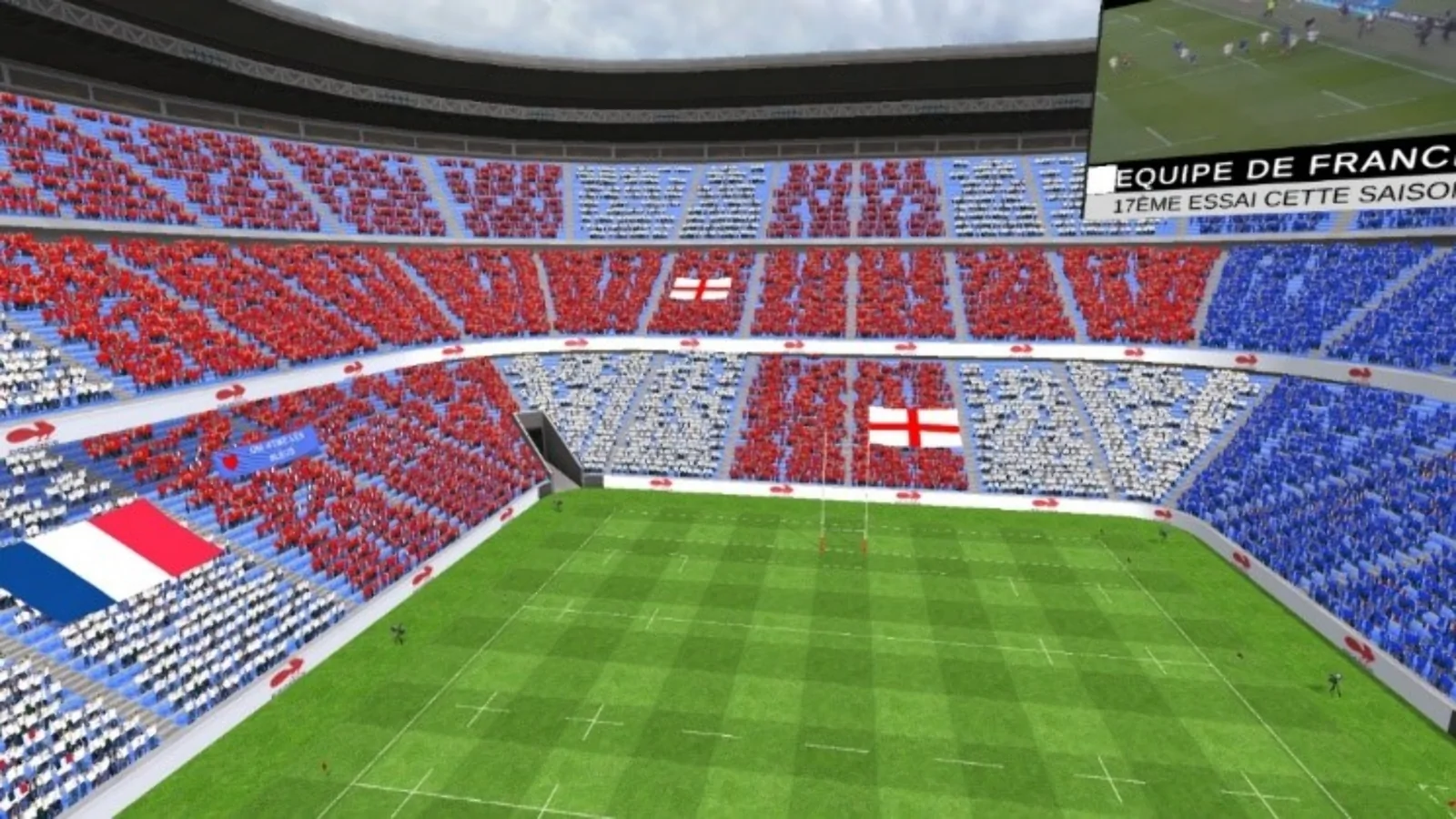
Stadium in the virtual environment. Virtual stadium displayed in the VE showing the colours and flags of the national French and English rugby teams (these two teams are an example, the colours and flags are adaptable to any rugby team).

A real-life video clip was integrated into the VE and projected onto the virtual stadium’s large screen, reinforcing participants’ immersive experience by simulating the moment they scored a try. This scene was designed to reproduce the sequence of events that are really happening on a rugby field during a game: the player scores a try, the stadium’s large screen then shows the name of the team who just scored, a replay of the action is displayed, the stadium announcer says the name of the team who just scored, the teammates arrive running at the player who scored and congratulate him. Since we worked with different clubs, the colours and flags of the teams in opposition were customised depending on the club of the participant. For this experiment, we worked with participants from three different French rugby clubs. For illustration and to keep the anonymity of our participants, Fig 1. shows a derby between France and England. Actually, for each club, the video displayed was chosen depending on the colours of the team and of their derby opponent, the videos were all showing a try with a clear action from one player seen from a drone view. That way, the participant could identify himself with the player scoring the try and have the illusion that he himself just scored for his team.

#### Avatar voices.

After the participant scored the try and saw the replay of it on the stadium’s screen, his virtual teammates came to congratulate him; the cheering animations were taken from the Final IK package in Unity and the library basic motions by Kévin Iglesias. The voices were human voices recorded with two researchers, with experience in team sports, involved in the research programme. The script was written in collaboration with a rugby player, and revised by the research team. We asked him to write down expressions typically used by players during a game or on a rugby field. After the congratulations of the avatars, the participant was transported to the centre of the field, with his virtual teammates, forming a concentration huddle (as it happens during a real game). Here again we asked the rugby player to write down the sentences a captain would say to his team in such a context. We recorded these sentences and then mixed them in Audacity (v. 2.4.2; [39]) to obtain the most realistic rendering.

#### Stadium sounds.

The surrounding sounds of the stadium were composed using several sources mixed in Audacity. One source was a recording taken at the “Atlantic stadium” in Bordeaux, during the France vs Georgia game, played in the 2021 Autumn Nations Series. Another source was a free of rights cheering sound from YouTube, and for each team we used a specific audio source in which supporters singing the team’s songs could be heard (e.g., for the French team: supporters singing the national anthem “La Marseillaise”).

### Measurements and material

One of the main challenges was to integrate questionnaires directly within the VE, using the transparent bubble effect (described just hereafter) in order to maintain high-quality immersion. This required simple, efficient measures, limiting the use of lengthy questionnaires. For several assessments, we used visual analogue scales anchored from 0 to 100, as well as 9 × 9 grids. This choice was made to ensure high sensitivity [40] and to avoid reproducibility bias in repeated measurements [41]. Our method proposes the integration of visual analogue scales within a VE, and allows measuring body ownership illusion, place illusion, plausibility illusion, and co-presence, while being immersed in the VE and without breaking presence for the participants to indicate their sensations without getting out of the VR application.

#### Participant characteristics.

Rugby level was defined as the player’s competitive status, categorised as either professional or aspiring professional. VR experience was assessed using a Likert-type scale ranging from 1 (never) to 7 (very often), based on the instruction: “Have you ever had any VR experiences?”.

#### Measures of identity.

Personal identity and social identity were measured with two scalar and a bipolar visual analogue scales. Indeed, through identity complexity, we conceptualized identity levels as independent, and thus with the possibility of covariation [20,21]. Measuring identity only with a bipolar scale would not have informed us about this dynamic. On the contrary, measuring identity only with two scalar scales would not specifically inform us about the relationship between the two identity levels. Therefore, we used both tools to assess identity. Inspired by previous work using the mouse paradigm to study emotion and identity [5,42], the first scalar visual analogue scale measured social identity (“In the present moment, I feel like a member of my team”), anchored from “not at all” to “completely”. The second visual analogue scale measured personal identity (“In the present moment, I feel like an individual, different from the members of my team”), anchored from “not at all” to “completely”. Finally, inspired by Gérat et al.’s [43] work, the last bipolar visual analogue scale measured personal identity and social identity (BId; “In the present moment I feel:”), differently anchored from −100 (“More like an individual, different from the members of my team”) to 100 (“More like a member of my team”). The visual analogue scale was a two-meter-long bar, and the text of the question appeared on top of this bar. On each extremity of the bar, a white label indicated the corresponding anchor statement. In the middle of the bar, a small yellow sphere acted as a cursor. Participants were asked to move the cursor, from the middle of the scale towards one anchor statement or the other, by touching it with their virtual hand. Fig 2 displays participants view when answering the visual analogue scale measuring their place illusion level.

**Fig 2:**
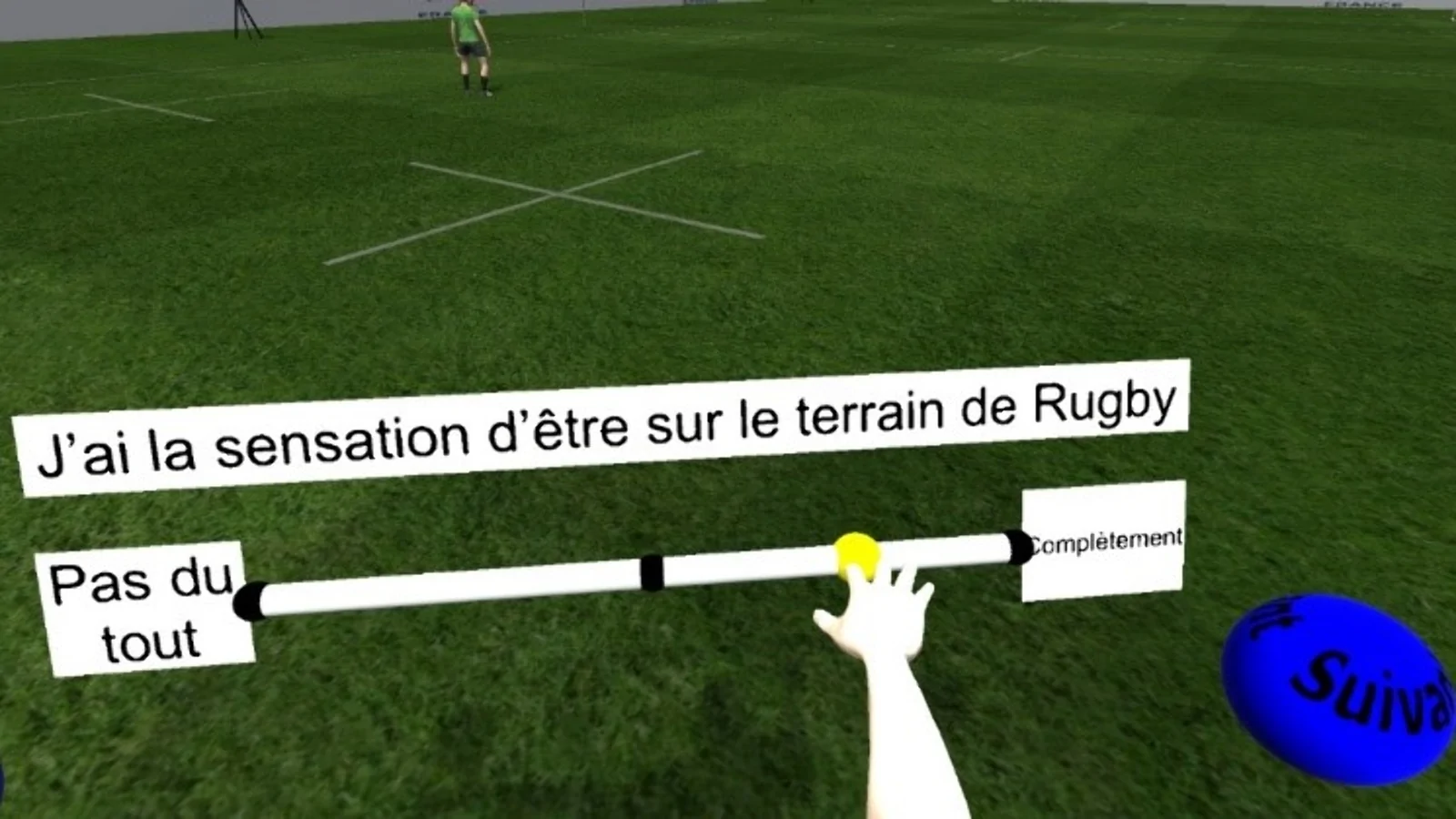
Visual analogue scale integrated in the virtual environment. The participant indicating the level of place illusion they feel, using their virtual hand to move the cursor (yellow sphere) on the visual analogue scale. On the right side of the participant, the button “suivant” (“next”) allows to step to the next question.

#### Measures of affects.

Affects were measured using Russel et al.’s [10] single measure grid. This 9 × 9 grid enables a simultaneous assessment of arousal, vertically anchored from −4 (“extreme sleepiness”; bottom) to +4 (“extremely high arousal”; top); and valence, horizontally anchored from −4 (“extremely unpleasant feelings”; left) to +4 (“extremely pleasant feelings”; right). The centre cell represents 0, the neutral point. Participants interacted with the grid in the same way as they did with the virtual visual analogue scale, by moving the yellow cursor on the two edges. Fig 3 is a screenshot of the use of this visual analogue grid (without the bubble effect).

**Fig 3:**
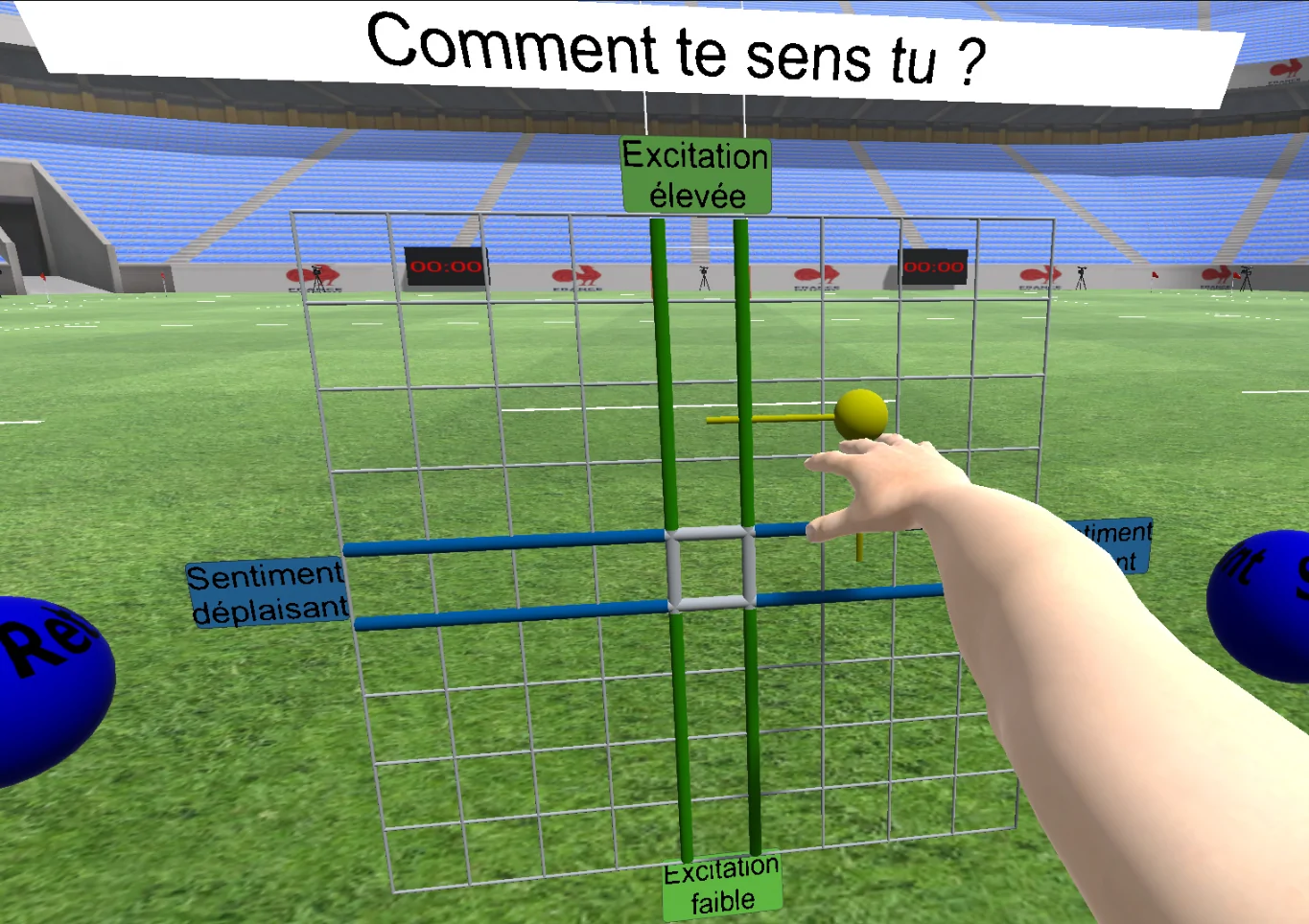
9  ×  9 grid integrated in the virtual environment. The participant indicating the valence and arousal of their affective state (without the bubble effect).

#### Measures of body ownership illusion, place illusion, plausibility illusion, and co-presence.

The question about body ownership illusion was taken from the standardized embodiment questionnaire by Gonzalez-Franco & Peck [44]: “When I look down, I feel that the body I am seeing is my own body”. The questions for measuring place illusion, place illusion and co-presence were adapted from Neyret et al. [45]. One modification was applied to all the questions: instead of using a Likert scale (ranging from −3 to +3), visual analogue scales were used. The place illusion visual analogue scale asked: “I have the sensation of being on the rugby field”. The place illusion visual analogue scale asked: “I have the sensation that I just scored a try”. Finally, the co-presence visual analogue scale asked: “I have the sensation of being on the rugby field with my teammates”.

We did not use any questionnaire related to simulator sickness given that the participants did not need to navigate inside the VE. We informed the participants that they could stop the procedure at any time, while being immersed in the VE, if they would experience any kind of cybersickness, headache or any other inconvenient sensation. No side effects of virtual reality were reported at the end of the experiment.

#### Transparent bubble technique.

To allow the participants to answer the questionnaires without extracting them from the immersion, we implemented a technique affecting the sound and image perceived in the VE. We called this technique the “transparent bubble” because it gives the participants the impression that they are in a soap bubble, transparent but affecting the perception of any object located outside this bubble. Several mechanisms are involved in the “transparent bubble” technique: darkening of the surrounding environment, toning down the surrounding sounds, and changing the depth of field (for technical details, see explanations in S1 Text). A measure (e.g., a visual analogue scale) appeared directly in 3D, sixty centimetres in front of them, at a height of 1.4 metres, inside the transparent bubble. The position of the visual analogue scale bar or grid centre was calculated depending on the height and position of the participants. The aim was to facilitate the interaction of participants with the measure and to allow them to position a cursor in the grid, or move a cursor on an axis, with a simple hand movement, as can be seen in Fig 2.

### Experimental procedure

All participants took part in a within-subjects design. Thus, each participant saw both collective and individual conditions. The VR system was installed directly onsite, at the participants’ training centres. Participants were first asked to read the complete information about the experimental procedure, including the exclusion criteria, and signed the corresponding informed consent. Participants were able to ask questions before the protocol, however all information related to the goal of the experiment was explained only after the end of the experimental procedure. As well, after doing the experiment, participants declared and signed that they would not discuss the content of the procedure with their teammates. That way, we made sure that all participants remained naïve about the goal of the experiment until the end of the protocol.

Participants filled in a demographic questionnaire. After that, participants were equipped with the HMD and the trackers, and then immersed in the VE. They first received audio instructions telling them to look at their body and move their arms and legs, to create embodiment in their virtual avatar. In this embodiment phase, they were standing on the virtual field and could see the reflection of their virtual avatar in a mirror placed in front of them on the field. The terrace seats were empty, and participants were standing alone on the field.

#### Pre-measures.

After the embodiment scene, participants had to answer a series of questions using the “interactive visual analogue scale” (see Transparent bubble technique). The visual perception of the rugby field was then blurred; however, participants could clearly see their virtual body when looking down (see Body ownership illusion, place illusion, plausibility illusion, and co-presence).

#### Virtual environment scenario.

After answering these questions, participants heard audio instructions telling them that they just scored a try and that they would be “transported” just after this try. The VR scene lasted two minutes. Two different VE scenarios corresponded to the Coll. or Indiv. conditions. For the Coll. scenario, the participant was first standing inside the in-goal area of the opponent (in the position where he was supposed to have scored), surrounded by supporters of both teams (wearing the colours of their respective team), and they could see the replay video of their try on the giant screens of the stadium. The participant could hear the stadium speaker shouting in the microphone that their team just scored a try (Stadium Speaker: “Try scored by *name of the team*”). the supporters wearing their team colours, were holding signs on which the name of the team was written (e.g., “GO *name of the team”* and “We love *name of the team”*). After seeing the video replay of their try, that lasted six seconds, the participant could see the opponents going towards their “in goal” area, and his teammates coming to congratulate him (see S3 Fig). His virtual teammates were surrounding and cheering him up for fifteen seconds and then running towards the centre of the field to meet in a concentration huddle, as the rugby players usually do on the field during a game. The team captain gave a brief motivational speech congratulating the team, and asking them to stay focus. His teammates were wearing the colours of the club and the opponents were wearing the colours of the opposed club, holding signs with the name of the team and singing the team’s anthem. On the giant screen the participant could see the logo and the name of their team. For the Indiv. scenario, participants could see the replay video of their try on the giant screens of the stadium as well. The participant could hear the stadium speaker shouting in the microphone that they just scored a try (Stadium Speaker: “Try scored by *name of the participant*”). The participant was standing alone inside the in-goal area of the opponent. The teammates were indifferent to him but the supporters held signs on which his name was written (e.g., “GO *name of the participant”* and “We love *name of the participant”*) and cheered him up. On the giant screen there was a picture of the participant together with his name instead of the team’s logo and team’s name. He was then transported in the middle of the field; no concentration huddle was made. Supporters, teammates and opponents were wearing neutral colours. The supporters held signs with the name of the participant and cheered him up. On the giant screen there was a picture of the participant instead of the team’s logo.

#### Post-measures.

After this two-minute scene, participants stayed on the rugby field and had to answer the visual analogue scale for the following variables: body ownership illusion, place illusion, place illusion, and co-presence.

### Data analysis

#### Main effect.

To evaluate the effect of the VR tool on identity levels (bipolar and scalar scales of social identity and personal identity), affects (valence and arousal), and immersion (body ownership illusion and place illusion), repeated measures ANOVA were conducted with pre- and post-measures across the Coll. and Indiv. conditions. Bonferroni corrections were applied to account for multiple comparisons. Furthermore, in order to evaluate the immersion, two tailed within-subject mean comparisons tests between post-measures of place illusion and co-presence across Coll. and Indiv. conditions were conducted. Threshold for significance is p = 0.05. Due to ecological constraints and the difficulty to recruit high-level rugby players, we conducted a post hoc power analysis using G-power for a repeated measures ANOVA (2 groups × 2 measure times). Entered parameters were an alpha of .05, a medium effect size (f = 0.25), and this study’s population (N = 40). The results indicated a power of 0.87.

#### Covariables.

We considered the influence of VR experience on the evolution of the main variables through pre and post measures and considered it as a covariable. In addition, the experience of professional games could potentially affect the VR experience as participants could have played in the Stade de France, and are more used to very crowded games. Thus, we also considered rugby level as a covariable. To evaluate the effect of the covariables on the evolutions of main effects variables, we employed Linear Mixed Effects (LME) models [46]. All analyses were conducted using the R package lme4 ensuring robust and reproducible results. Each model predicted the dependent variable from the interaction between Time and rugby level, and the interaction between Time and VR experience, with participant ID included as a random intercept.

## Results

The means, standard deviations and correlations between all the included variables are presented in Table 1. In addition, Fig 4 displays descriptive graphics of the obtained results. LME models are displayed in S4 Table in supplementary material. Only significant results are described below.

**Table 1:** Means and standard deviations of conditions.

	VR	RL	SId	PId	BId	Vlc	Arsl	BOI	PI	Psl	CoP
**General**											
**Mean**	1.62	1.40									
**Standard Deviation**	1.28	0.497									
**Collective condition pre-measures**											
**Mean**			73.1	45.3	67.1	77.3	64.6	62.9	69.8		
**Standard Deviation**			21.3	25.6	29.4	13.8	21.7	25.4	18.8		
**Collective condition post-measures**											
**Mean**			83.1	31.9	82.9	87.5	79.5	70.2	80.3	67.1	80.3
**Standard Deviation**			17.7	27.8	20.7	9.37	21.0	22.5	17.1	26.9	17.6
**Individual condition pre-measures**											
**Mean**			61.3	49.6	62.3	72.9	65.6	62.0	70.1		
**Standard Deviation**			27.7	28.6	27.1	15.2	22.6	25.7	19.9		
**Individual condition post-measures**											
**Mean**			69.2	52.6	61.0	75.7	72.9	62.1	75.1	67.0	59.3
**Standard Deviation**			30.5	28.2	32.0	23.8	25.7	27.7	19.0	23.3	26.4

*Note.* *** *p* < .001; ** *p* < .01; * *p* < .05; VR = VR experience; RL = Rugby level; SId = Social identity; PId = Personal identity; BId = Binary measure of social and personal identity; Vlc = Affective valence; Arsl = Affective Arousal; BOI = Body ownership; PI = Place illusion; PsI = Plausibility illusion; CoP = Co-presence.

**Fig 4:**
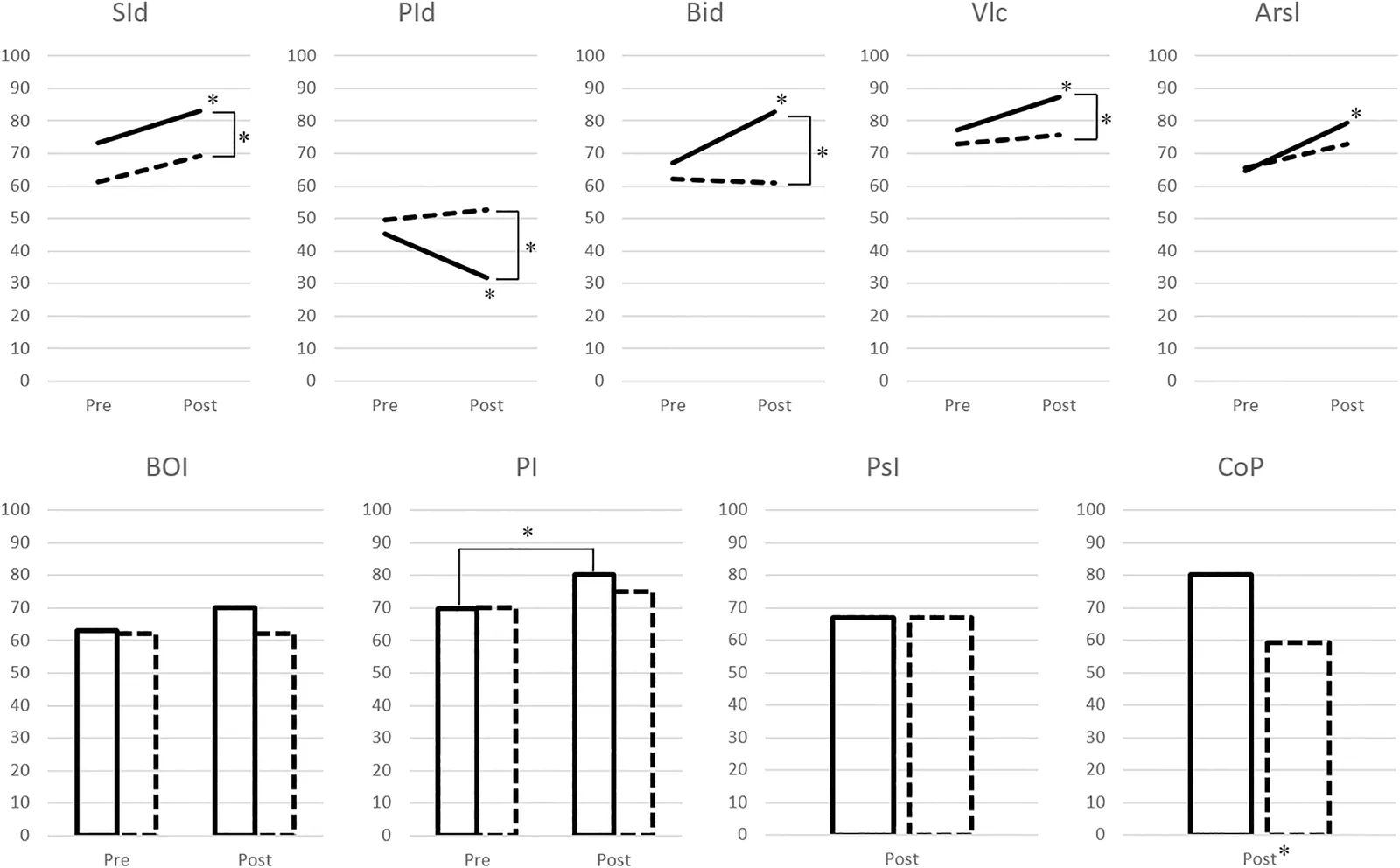
Graphical representation of the evolution of study variables across conditions. Solid lines refer to Coll. condition and dotted lines refer to Indiv. condition. * on a line = Significant difference between pre and post; * on a bracket = significant difference between two conditions; SId = Social identity; PId = Personal identity; BId = bipolar scale of identity; Vlc = Affective valence; Arsl = Affective arousal; BOI = Body ownership; PI = Place illusion; PsI = Plausibility illusion; CoP = Co-presence; Coll. = Collective condition; Indiv. = Individual condition.

### Identity induction

#### Social identity activation.

Fig 5 presents the data concerning the evolution of social identity levels in the collective and individual conditions. Social identity level increased after the exposure to the VR scene in the collective condition (Median pre = 75.56 – Median post = 86.19). There was no difference in social identity levels after the exposure to the VR scene in the individual condition (Median pre = 67.65 – Median post = 72).

**Fig 5:**
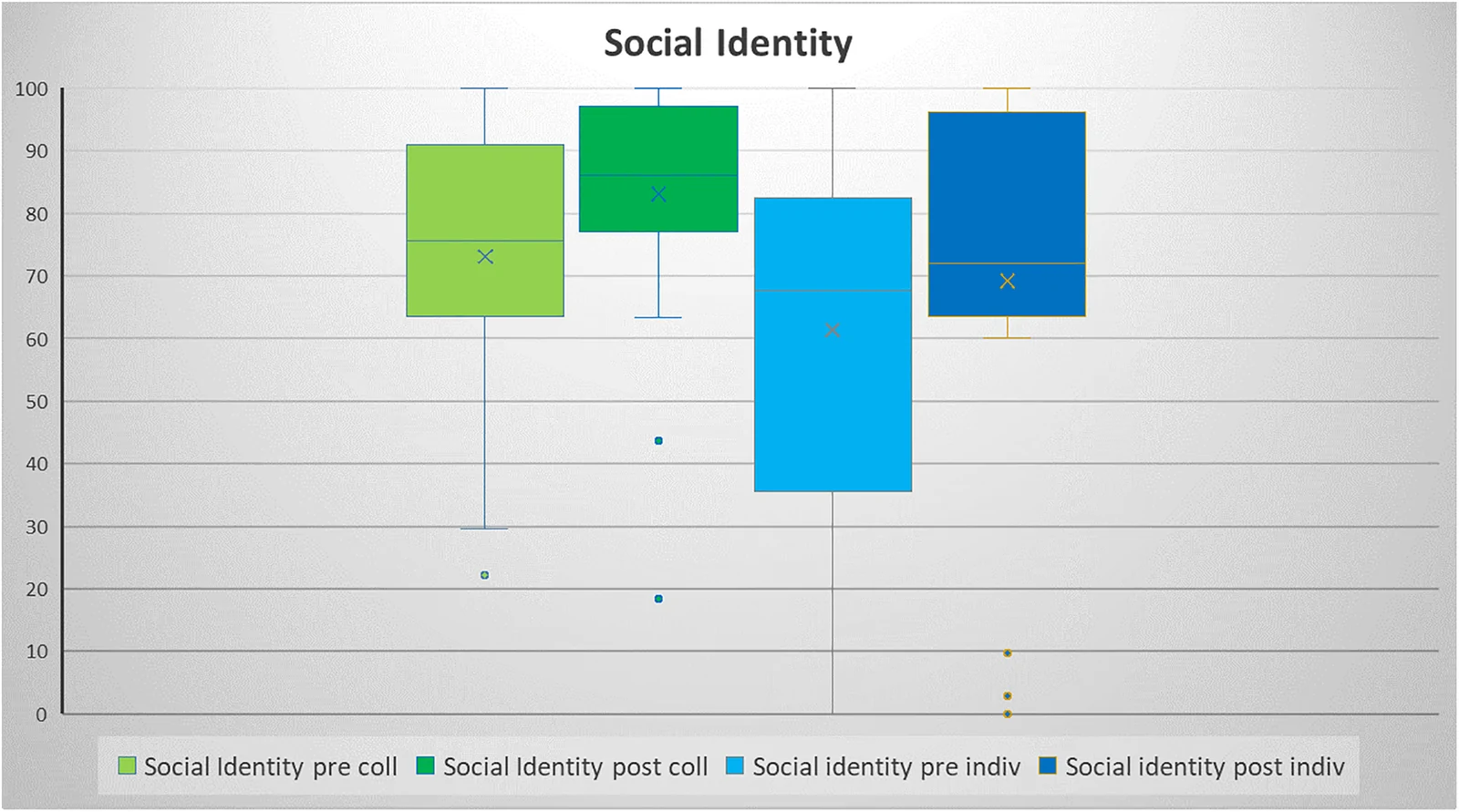
Social identity levels before and after exposure to the virtual environment, in both conditions. Box plot presenting the level of social identity before (Social Identity pre) and after (Social Identity post) the exposure to the try scene in the collective (coll) and individual (indiv) conditions. The horizontal thick lines indicate the value of the medians, the boxes are the interquartile ranges (IQR), the whiskers extend from maximum (median – 1.5*IQR, smallest value) to minimum (median + 1.5*IQR, largest value). Outliers are shown individually as separated dots.

In order to examine social identity induction, data were first analysed using a 2 (Condition: Coll. vs. Indiv.) × 2 (Time: Pre- vs. post-measure) ANOVA, with repeated measures on the last factor. The analysis revealed a significant main effect of Condition F(1,34) = 12.92, p = 0.001, η²_p_ = 0.275; as well as of Time, F(1,34) = 7.34, p = 0.01, η²_p_ = 0.178.

After applying a Bonferroni correction, post hoc tests indicated a significant difference between the two conditions at post-measure, t(34) = 2.93, p = 0.036. Social identity showed a significant difference between pre-measure (M = 73.1; SD = 21.3) and post-measure (M = 83.1; SD = 17.7) in the Coll. condition, t(34) = −3.40, p = 0.01, corresponding to an increase of 13.68%. Using LME, rugby level and VR experience did not significantly predict baseline social identity levels scores, nor did they moderate the pre–post change. The evolution from pre to post was therefore not explained by individual differences in sport expertise or VR familiarity. The pattern of means, as presented in Fig 4, partially supports the study hypotheses.

#### Personal identity activation.

Fig 6 presents the data concerning the evolution of personal identity levels in the collective and individual conditions. Personal identity level decreased significantly in the collective condition (Median pre = 38.32 – Median post = 28.5), whereas personal identity level increased significantly in the individual condition (Median pre = 42.23 – Median post = 56.50).

**Fig 6:**
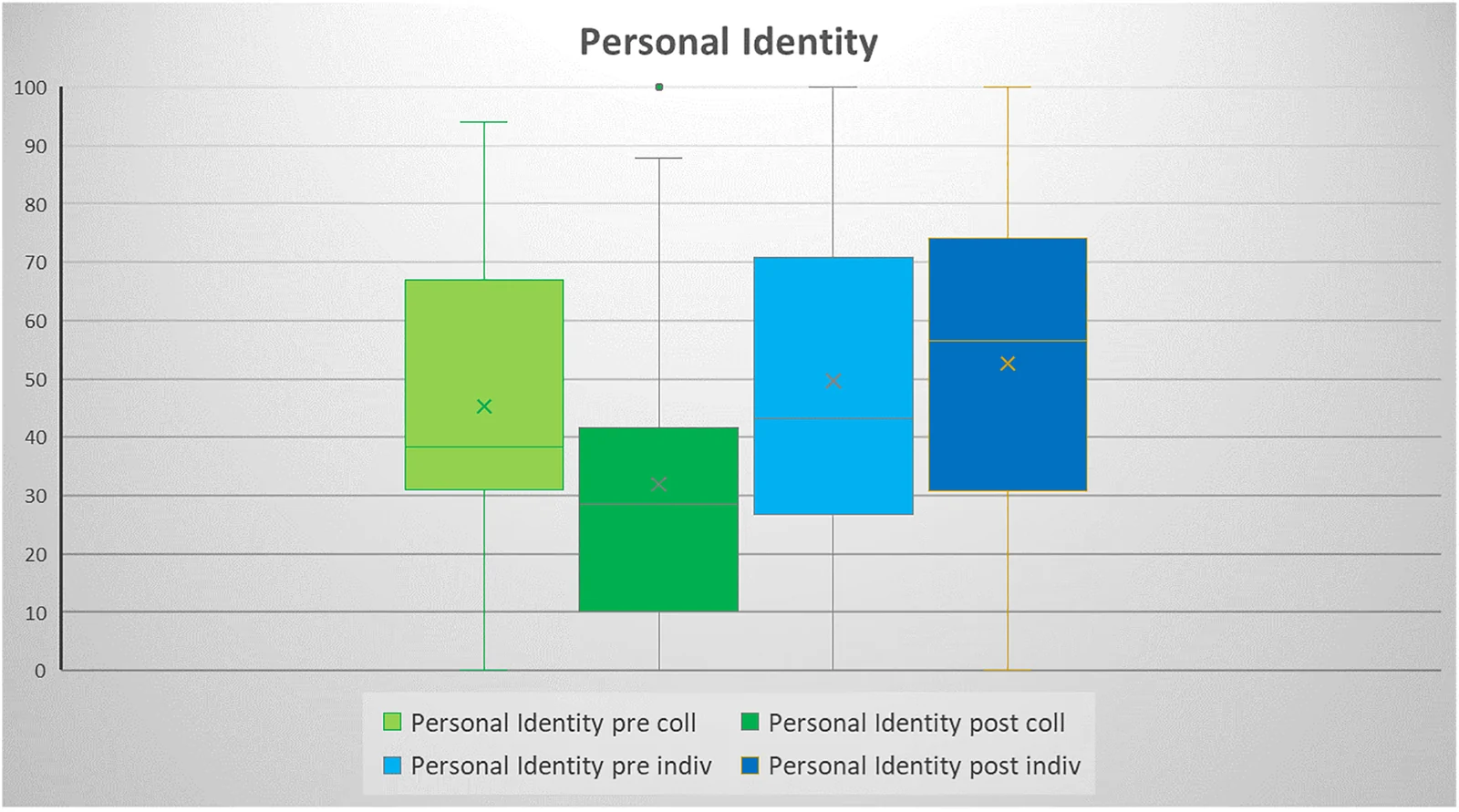
Personal identity levels before and after exposure to the virtual environment, in both conditions. Box plot presenting the level of personal identity before (Personal Identity pre) and after (Personal Identity post) the exposure to the try scene in the collective (coll) and individual (indiv) conditions. The horizontal thick lines indicate the value of the medians, the boxes are the interquartile ranges (IQR), the whiskers extend from maximum (median – 1.5*IQR, smallest value) to minimum (median + 1.5*IQR, largest value). Outliers are shown individually as separated dots.

A repeated measure ANOVA was conducted on personal identity, which showed a significant main effect of Condition F(1,34) = 9.65, p = 0.004, η²_p_ = 0.221. This main effect was further qualified by a significant Condition × Time, F(1,34) = 4.87, p = 0.034, η²_p_ = 0.125.

Post hoc tests indicated a significant difference between the two conditions at post-measure, t(34) = ‑3.14, p = 0.021. Personal identity showed a significant difference between pre-measure (M = 45.3; SD = 25.6) and post-measure (M = 31.9; SD = 27.8) in the Coll. condition, t(34) = 2.81, p = 0.049, corresponding to a decrease of 29.58%. LME indicated that neither rugby level nor VR experience had a significant effect on baseline scores, nor did they moderate the pre–post change in personal identity.

#### Identity dominance (measured via bipolar scale).

Fig 7 presents the data concerning the evolution of identity levels in the collective and individual conditions. Social identity level, measured with a binary choice, increases significantly in the collective condition (Median pre = 72.91 – Median post = 89.18), but it did not evolve much in the individual condition (Median pre = 63.55 – Median post = 68.84). A repeated measure ANOVA was conducted on BId, which showed a significant main effect of Condition F(1,34) = 8.63, p = 0.006, η²_p_ = 0.202; as well as Time, F(1,34) = 5.85, p = 0.021, η²_p_ = .147. This main effect was further qualified by a significant Condition × Time, F(1,34) = 7.45, p = 0.010, η²_p_ = 0.180. Post hoc tests indicated a significant difference between the two conditions at post-measure, t(34) = 4.53, p < 0.001. BId showed a significant difference between pre-measure (M = 67.1; SD = 29.4) and post-measure (M = 82.9; SD = 20.7) in the Coll. condition, t(34) = −4.12, p = 0.001, corresponding to an increase of 23.55%. LME indicated that neither rugby level nor VR experience had a significant effect on baseline scores, nor did they moderate the pre–post change in identity dominance.

**Fig 7:**
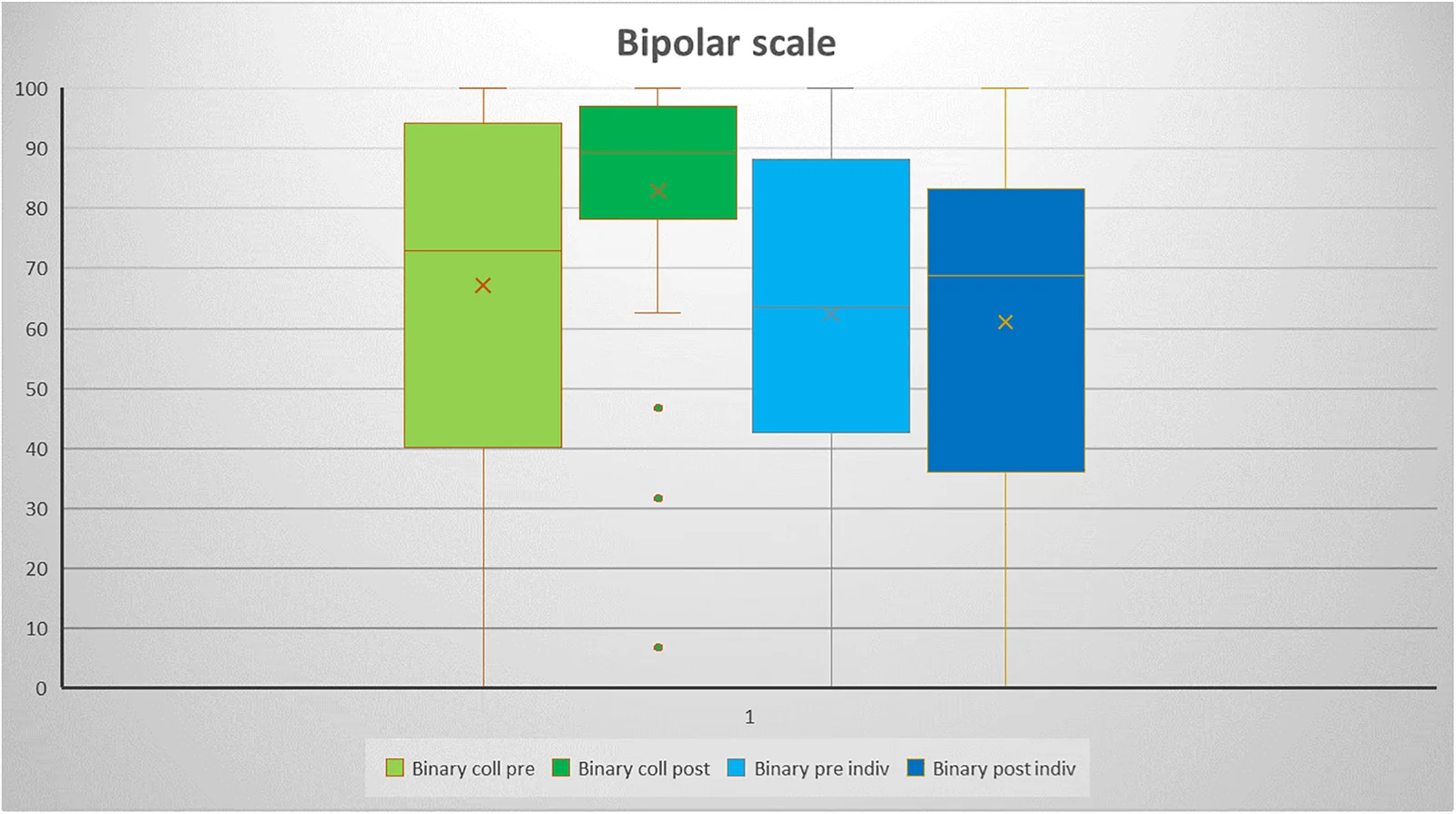
Identity levels as bipolar before and after exposure to the virtual environment, in both conditions. Box plot presenting the level of social identity (with a binary choice between social and personal identity) before (Binary pre) and after (Binary post) the exposure to the VR scene in the collective (coll) and individual (indiv) conditions. The horizontal thick lines indicate the value of the medians, the boxes are the interquartile ranges (IQR), the whiskers extend from maximum (median – 1.5*IQR, smallest value) to minimum (median + 1.5*IQR, largest value). Outliers are shown individually as separated dots.

### Affective induction

#### Valence.

A repeated measure ANOVA was conducted on valence, which showed a significant main effect of Condition F(1,34) = 10.59, p = 0.003, η²_p_ = 0.237; as well as Time, F(1,34) = 12.66, p = .001, η²_p_ = 0.271.

Post hoc tests indicated a significant difference between the two conditions at post-measure, t(34) = 3.08, p = 0.024. Valence showed a significant difference between pre-measure (M = 77.3; SD = 13.8) and post-measure (M = 87.5; SD = 9.37) in the Coll. condition, t(34) = −4.61, p < .001, corresponding to 13.20%. LME indicated that neither rugby level nor VR experience had a significant effect on baseline scores, nor did they moderate the pre–post change in valence.

#### Arousal.

A repeated measure ANOVA was conducted on arousal, which showed a significant main effect of Time F(1,34) = 12.73, p = 0.001, η²_p_ = 0.272.

Post hoc tests indicated a significant difference between pre-measure (M = 64.6; SD = 21.7) and post-measure (M = 79.5; SD = 21.0) in the Coll. condition, t(34) = −5.62, p < .001, corresponding to of 23.07%. LME indicated that neither rugby level nor VR experience had a significant effect on baseline scores, nor did they moderate the pre–post change in arousal.

### Illusions in virtual reality

#### Body ownership.

Fig 8 presents the data concerning the evolution of body ownership illusion levels in the collective and individual conditions. A repeated measure ANOVA was conducted on body ownership illusion, which showed no significant main effect nor interaction. Post-hoc test also revealed no significant differences. LME indicated that VR experience had no significant effect on baseline scores, nor did rugby level and VR experience moderate the pre–post change in arousal. However, rugby level had a significant effect on baseline scores. Indeed, professional players had significantly lower BOI than aspiring professional players (β = −18.831; p = 0.025).

**Fig 8:**
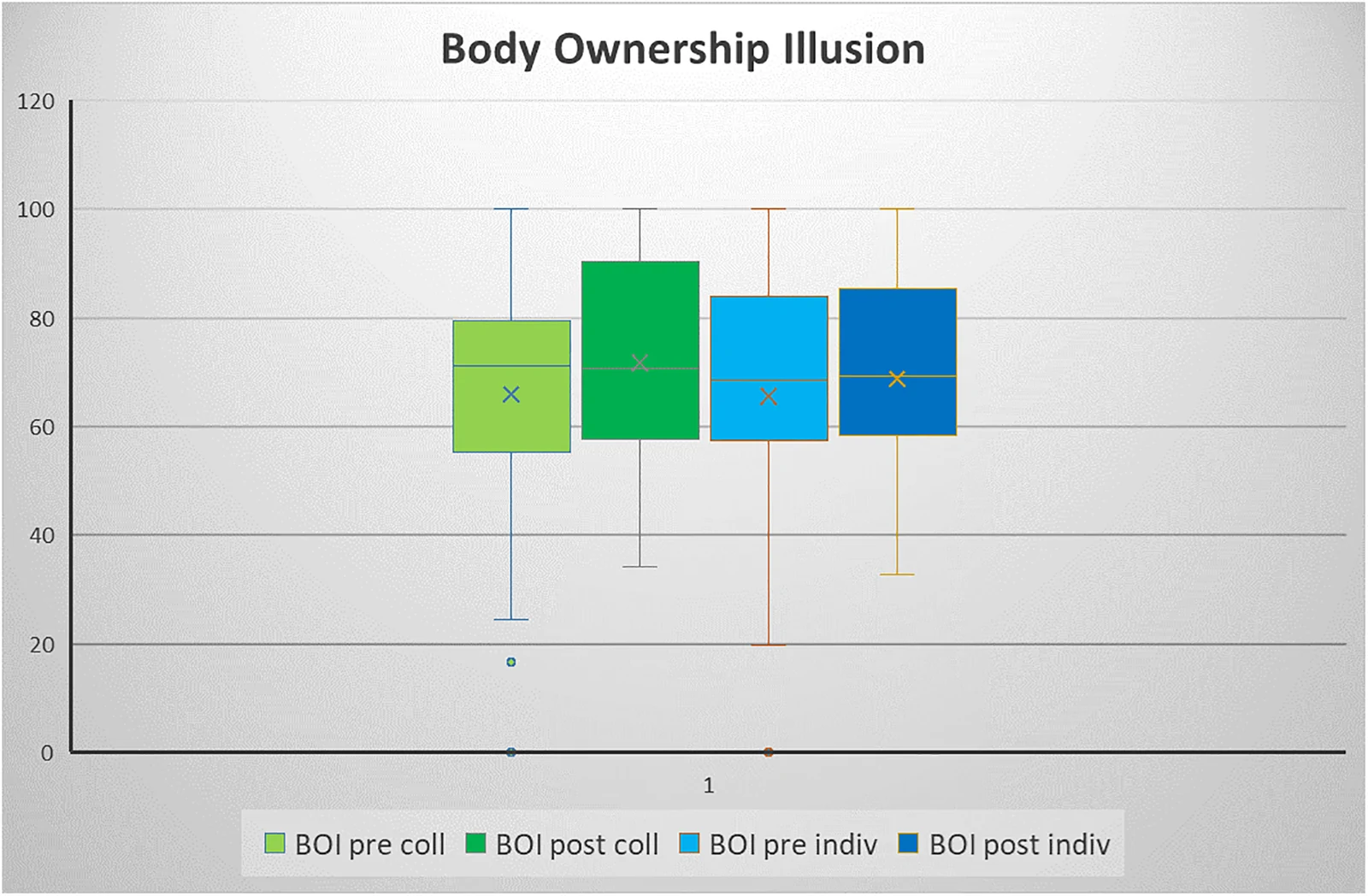
Body ownership before and after exposure to the virtual environment, in both conditions. Box plot presenting the level of body ownership illusion before (BOI pre) and after (BOI post) the collective (coll) and the individual (indiv) condition. The horizontal thick lines indicate the value of the medians, the boxes are the interquartile ranges (IQR), the whiskers extend from maximum (median – 1.5*IQR, smallest value) to minimum (median + 1.5*IQR, largest value). Outliers are shown individually as separated dots.

#### Place illusion.

Fig 9 presents the data concerning the evolution of place illusion levels in the collective and individual conditions. In both conditions, place illusion was already high before the exposure to the try scene in VR, it got higher in both conditions but the increase was more important in the collective condition (place illusion pre coll Median = 68.30, place illusion post coll Median = 81.2) – (place illusion pre indiv Median = 69.8, place illusion post indiv Median = 72.2). This means that the social interaction with the avatars, who were coming towards the participant to congratulate him after the try, increased his sense of being there on the rugby field.

**Fig 9:**
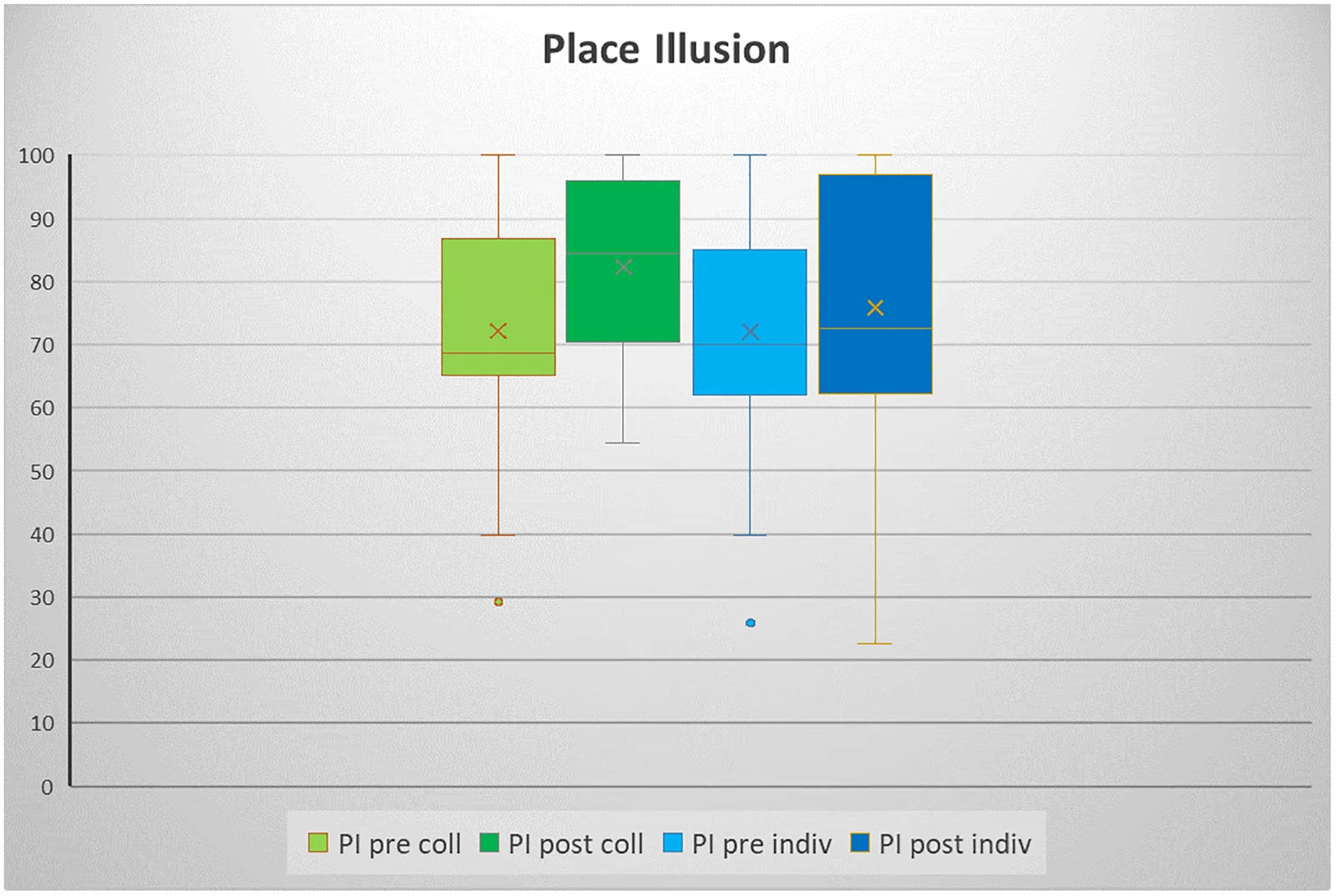
Place illusion before and after exposure to the virtual environment, in both conditions. Box plot presenting the level of place illusion before (PI pre) and after (PI post) in the collective condition (coll) and in the individual (indiv) condition. The horizontal thick lines indicate the value of the medians, the boxes are the interquartile ranges (IQR), the whiskers extend from maximum (median – 1.5*IQR, smallest value) to minimum (median + 1.5*IQR, largest value). Outliers are shown individually as separated dots.

A repeated measure ANOVA was conducted on place illusion, which showed a significant main effect of Time F(1,34) = 11.76, p = 0.002, η²_p_ = 0.257. Post hoc tests indicated a significant difference between pre-measure (M = 69.8; SD = 18.8) and post-measure (M = 80.3; SD = 17.1) in the Coll. condition, t(34) = −3.36, p = 0.012, corresponding to of 15.04%. LME indicated that VR experience had no significant effect on baseline scores, nor did rugby level and VR experience moderate the pre–post change in arousal. However, rugby level had a significant effect on baseline scores. Indeed, professional players had significant lower PI than aspiring professional players (β = −12.610; p = 0.038).

#### Plausibility illusion.

Fig 10 presents the data concerning place illusion levels in the collective and individual conditions. Levels of plausibility illusion were very high in the individual condition (median = 70.8) and in the collective condition -median = 68.3). A Wilcoxon mean comparison test was conducted on place illusion between both Coll. and Indiv. conditions. No significant differences were shown for place illusion (W = 312, p = 0.811). A simple linear model indicated that professional players had significant lower Psl than aspiring professional players (β = − 18.765; p = 0.004). In addition, participants with better VR experience had significant higher Psl than those with lower VR experience (β = 5.046; p = 0.046).

**Fig 10:**
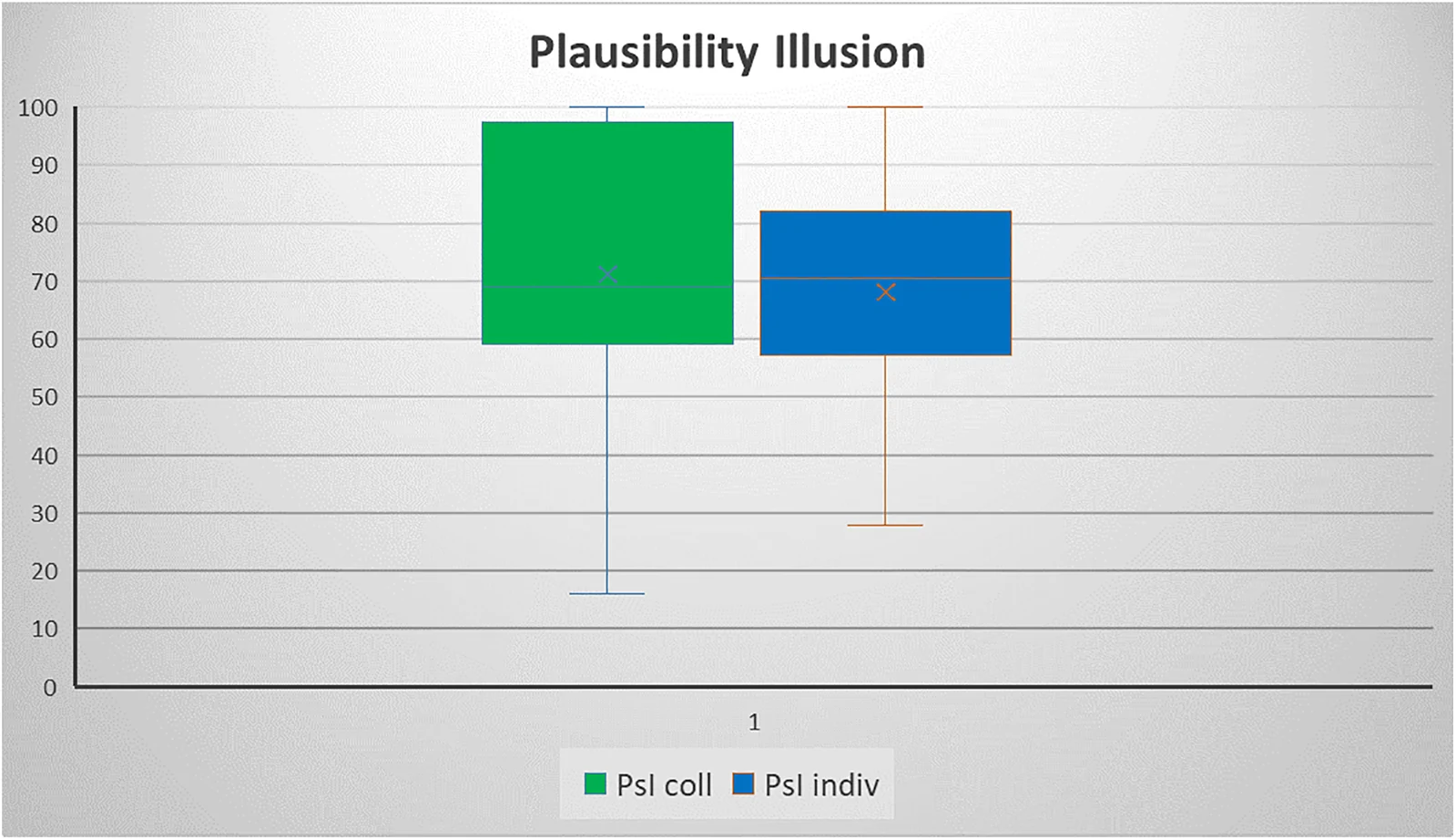
Plausibility illusion after exposure to the virtual environment, in both conditions. Box plot presenting the level of plausibility illusion after exposure to the try scene in the collective condition (PsI coll) and after the try scene in the individual condition (PsI indiv). The horizontal thick lines indicate the value of the medians, the boxes are the interquartile ranges (IQR), the whiskers extend from maximum (median – 1.5*IQR, smallest value) to minimum (median + 1.5*IQR, largest value). Outliers are shown individually as separated dots.

#### Co-presence.

Fig 11 presents the data concerning co-presence levels in the collective and individual conditions. Levels of co-presence were significantly higher in the collective condition (median = 80.25), in which the avatars came to congratulate the participant. In the individual condition the level of co-presence had a median of 61.20. A Wilcoxon mean comparison test was conducted on co-presence between both Coll. and Indiv. conditions. there was a significant difference between Coll. (M = 80.3; SD = 17.6) and Indiv. (M = 59.3; SD = 26.4) condition for co-presence (W = 494, p < .001), which means that co-presence was higher in the Coll. compared to the Indiv. condition. Even if the teammates were present on the rugby field in the individual condition, the level of co-presence was significantly higher when the virtual teammates came to congratulate the participant. A simple linear model indicated that neither rugby level nor VR experience had a significant effect on baselines scores.

**Fig 11:**
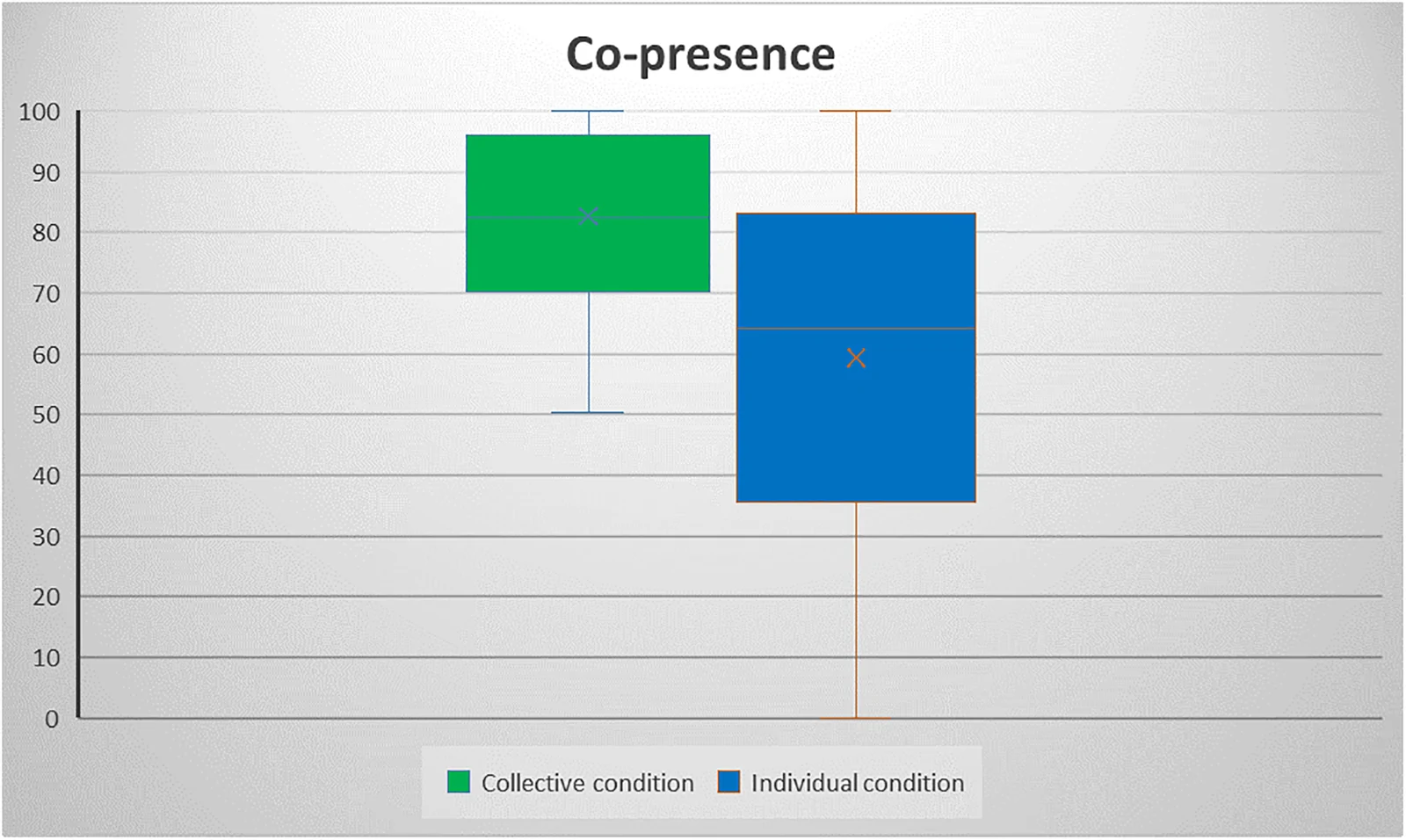
Co-presence after exposure to the virtual environment, in both conditions. Box plot presenting the level of co-presence after exposure to the try scene in the collective condition, and after the try scene in the individual condition. The horizontal thick lines indicate the value of the medians, the boxes are the interquartile ranges (IQR), the whiskers extend from maximum (median – 1.5*IQR, smallest value) to minimum (median + 1.5*IQR, largest value). Outliers are shown individually as separated dots.

## Discussion

The aim of this study was to test a virtual reality (VR) method to induce and measure affective states in rugby players using the framework of identity processes. We hypothesised that, following immersion in the virtual environment (VE) under the collective condition (Coll.), participants would report higher levels of social identity, physiological arousal, and pleasant affect, as well as greater co-presence compared to the individual condition. Conversely, we expected that immersion in the individual condition (Indiv.) would lead to an increase in personal identity salience, accompanied by lower levels of arousal and pleasant affect. Finally, we anticipated that core components of immersive experience—namely, place illusion, plausibility illusion, and body ownership illusion—would remain stable across both conditions. Overall, the findings of the current study showed that the collective condition resulted in an increase of the social identity level, of the arousal, and of the pleasant affects. It also resulted in an increase of the level of Place Illusion, a result that we did not expect. Furthermore, it led to a decrease of the personal identity level. We also observed a higher level of co-presence in the collective condition than in the individual one.

In contrast, results show that there was no significant difference between pre- and post-measure**s** for any of the measured variables (personal and social identity measured through scalar and bipolar scales, affects’ valence and arousal, body ownership and place illusion) in the Indiv. condition. This means that the Coll. condition mainly provoked the desired effects, but not the Indiv. Condition; thus, partially validating our hypotheses. Similarly, immersion in the VE induced a change in social identity level in the Coll. condition, but not in the Indiv. condition, which also partially validates our hypothesis.

Also, the immersive interactive visual analogue scale method we developed in this study showed good effectiveness for measuring place illusion, place illusion, body ownership illusion, co-presence, identity and affective levels. While a break in place illusion could be expected when the participants see a floating visual analogue scale in front of them, our results show high levels of place illusion and place illusion, highlighting that no break of place illusion occurred.

### VR effect on affects and social identity

Regarding identity processes, our findings may suggest that the social cues embedded in the VE in the collective condition were effectively perceived by participants as conveying social value [12,13] — in particular, through symbols or images associated with their own clubs [47,48]. This result echoes current literature stating that pleasant affects are correlated with social identity [4,5,7]. Furthermore, the Indiv. condition did not have any effect on personal identity. One possible explanation is that participants did not appear to perceive any social cues that would enhance the perceived value of the self as individuals. This may also indicate that the intent to make participants feel like isolated individuals in the Indiv. condition (teammates with grey jerseys, no interaction with teammates, supporters holding signs with the participant’s name, etc.) did not increase personal identity.

This study’s results may also suggest that both identity levels are negatively correlated to each other, as predicted by Turner et al. [13]. Specifically, in the Coll. condition, as social identity increased between pre- and post-measure, personal identity decreased. However, when comparing social identity and personal identity levels in each of the condition, this study’s results support previous literature showing an independence of both identity levels [5], indicating that personal identity may not simply be the opposite of social identity. In the individual condition, no social cues seemed to convey added value to viewing oneself as an individual. Coherently and consequently, arousal did not increase in the Indiv. condition. As arousal is a core component of emotions [11,49], one can wonder if affects were triggered at all in the Indiv. condition. Previous literature has also reported mixed results [5,6,49,50]. In this sense, future research should consider including physiological measures such as heart rate and skin electrodermal activity to add multiple evidence regarding the presence or not of affects [51], as well as gaze tracking in order to measure attention correlated with identity saliency. In addition, cybersickness was not measured because our experimental procedure did not require navigation within the VR environment. Instead, participants were transported to different areas of the stadium using a fade to black. Future studies should nonetheless control for cybersickness, as such transitions may influence participants’ emotional and behavioural responses. In addition, cognitive style could also influence this study’s variables, and should be considered for future studies as well.

Another result deserves attention. Indeed, the bipolar visual analogue scale measuring identity (BId) displays the same results as the social identity visual analogue scale (see Fig 4). The results of the bipolar visual analogue scale confirm that the coll. condition triggered an increase in social identity level. More broadly, these results may suggest that social identity plays a central role in team sports. While identity levels are generally thought to rely on the same cognitive mechanisms—differing only in the locus of importance (i.e., group vs. individual) [12,13] —our findings may indicate that these levels may in fact operate differently. Specifically, only the collective condition led to changes across nearly all measured variables, including affective states, social identity, and VR-related variables such as body ownership and co-presence. This pattern not only aligns with previous research showing links between social identity and positive affect [4,6], but also with studies suggesting that social identity enhances athletes’ awareness of their emotional states [43]. Cues intended to increase identity saliency for both identity levels were designed to be symmetrical, with interactions with teammates for social identity and no interaction for personal identity. The social interactions were unfolding in the way that usually occurs when a try is scored during a real game. However, one could argue that “no interaction” is simply the absence of interaction, rather than its true symmetrical counterpart. Despite the need to keep the unfolding of events as realistic as possible, future research could therefore implement more explicitly structurally comparable stimuli and measure their impact on individual identity levels.

Moreover, social identity remained relatively high even in the individual condition, whereas personal identity levels did not vary significantly across conditions. This reinforces the idea that it is primarily changes in social identity—not personal identity—that influence athletes’ psychological experience in team sports. Based on our data, the most effective way to reduce players’ sense of group belonging during this experimentation appears to be to decrease social identity, rather than increasing personal identity. Consequently, athletes’ cognitive and affective experiences seem to be more influenced by variations in social identity than by changes in personal identity. These findings support prior research on social identity in sport [3,52] and on identity complexity [20,21,53], while also raising important questions about whether this primacy of social identity is specific to team sport contexts. To go further, this study would have benefited from the inclusion of post-test measures. However, because a substantial part of affective responses is tied to specific objects or events [54], and because the affects elicited by this protocol are linked to the identity level that was experimentally induced [8]—both of which are highly contextual—such post-test measures would need to be collected within a relatively short time frame after the protocol (one or two hours), and should not be administered after or during other events that participants may experience as personally significant. In addition, although this study’s identity measures are based on current literature [5,42,43] and are close to an existing validated measure [55], these measures themselves have not yet undergone psychometric validation. Future research should conduct comparative validation against established measures to confirm reliability and construct validity.

### VR effect on illusion and immersion variables

It is interesting to observe that our participants reported higher sensation of co-presence and place illusion in the collective conditions. In this scene, the virtual teammates came to congratulate the participant after he scored the try and then, were all united in a huddle, at the centre of the ruby field (as rugby players usually do after a significant try during a real rugby game). In this huddle time, the team captain was giving a motivation speech between the try and the conversion kick. All the sounds played during the try scene were real. The cheering and singing sounds of the supporters were recorded during a real rugby game; the voices of the avatars were human voices acting a script established in collaboration with a rugby player. Many of our participants reported that the voices of the avatars were very realistic, and that the words they were saying were actually very close to what is said on the rugby field in a real game context. We believe that this had a positive impact on the levels of place illusion and co-presence experienced by our participants. The only element of place illusion that we measured was the participant’s sensation of “just having scored a try”. It would have been interesting to measure the plausibility component of co-presence using the question. However, this question raises an issue with our interactive visual analogue scale method, because it cannot really be adapted to “in-the-moment measures”. Indeed, it is inconvenient to ask the participant about his interaction with the avatars while he is having this interaction, even using our “transparent bubble” method. This explains why, in this study, we used only the scale related to the place illusion component of co-presence relating to the sensation of being on the field with teammates. That way, participants could easily look at their teammates (blurred through the transparent bubble) and rate to which extent they felt they were on the field with them. The present tense used in all the questions had an interesting and unexpected effect: participants “tested” their ongoing sensations. For instance, they searched for their virtual teammates on the field before answering the co-presence question. As well, when reading the question related to body ownership, what most of them did, right away, was actually to look down at their body and test in real time the level of body ownership illusion they were experiencing in the moment. It should be noted that our results indicate that professional players reported lower levels of body ownership and place illusion compared to aspiring professionals. In this sense, research has shown that expert athletes outperform non-experts in body consciousness tasks [56], notably by using additional contextual cues beyond visual information while remaining highly sensitive to visual cues [57]. Another explanation could be that experts are more accustomed to this type of environment, such that even minor discrepancies between the VR scene and their real-world expectations may reduce these two variables’ scores.

The main difference between the collective and the individual conditions were the interactions with the virtual teammates, first coming to congratulate the participant after he scored the try, and then uniting in a circle in order to synchronize the respiration after the captain’s speech, between the scoring and the transformation. As stated in the introduction, place illusion corresponds to the subjective-phenomenal experience of “now being there” in reality [58]. It is interesting to note that place illusion increased with the presence of virtual teammates, as if the auditory perception of human voices synchronized with the presence of the virtual teammates on the virtual field would reinforce the subjective-phenomenal experience of being there, by it being confirmed by the fact that the space is shared with other human agents. As above-mentioned, cognitive and behavioural responses to a VE are based on the predictions that were learnt in previous interactions of an individual with the real environment [59–61]. Here we can argue that the participant’s interaction with the virtual teammates corresponded to the predictions of interactions with teammates on a rugby field, and therefore resulted in high levels of co-presence and place illusion. Another factor that can explain these results is that the collective condition was closer to what players actually experience on the rugby field in reality. Although the VR environment did not aim at reproducing the full complexity of a real rugby game, it was designed to recreate emotionally and socially meaningful sport situations while maintaining experimental control over identity-related cues. As seen in the introduction, behavioural responses to a constantly changing environment are based on the prior beliefs that were learnt in previous interactions of an individual with the environment [60]. In the individual condition the teammates are there on the field but they do not interact with the participant at all. It is interesting to note that the interventions of the virtual teammates and the higher co-presence did not have an impact on the plausibility illusion level. In both conditions, participants did have a strong sensation of having scored a try.

## Conclusions

This study presents early but promising insights into the use of virtual reality (VR) for exploring the link between identity and affects in a sport-specific setting. One of the most original aspects lies in the idea of using identity processes not just as a background variable, but as a lever to actively shape affective experience. The way we embedded the measurement tools directly into the VR setting—using what we referred to as the “Bubble technique”—allowed players to report on their affects without stepping out of the immersive experience. From a technical perspective, this is a meaningful advance for the use of VR in social sciences. From a practical one, it opens up realistic possibilities for coaches and psychologists to design affectively meaningful training environments. While more work is needed, especially across different sports and populations, this approach provides a solid basis for future research and real-world applications.

## Supporting information

S1 Text The transparent bubble. (DOCX)

S2 Fig Embodiment phase. The participant sees themselves in the virtual mirror placed in front of them, they receive audio instructions telling them to move their hands and legs.
(TIF)

S3 Fig Gathering of teammates. Virtual teammates coming towards the participant to congratulate them after they scored the try.
(TIF)

S4 Table LME and simple linear models assessing covariables influence. (DOCX)
