# Supplementary material for: Inducing and measuring identity levels in professional rugby players during immersion in an interactive virtual environment
Source: PLoS One. 2026 Oct 7;21(10):e0359964. doi: 10.1371/journal.pone.0359964 (PMC13645085; doi:10.1371/journal.pone.0359964)
Supplement: Supplementary file 1 [file pone.0359964.s001.docx]

**Supporting information 1. The transparent bubble**

**The transparent bubble effect**

For the darkening effect, a 3D model of a sphere with a one-meter radius was placed around the head of the participant. Normal orthogonal vectors were oriented from inside the sphere towards the internal side of the sphere*.* We used the standard Unity material (transparent) with a black value for the albedo colour of the standard material of the object, and with an alpha value varying between 0 and 50%. The bubble was always placed around the head of the participant, however the visual effect appeared only when the value of 50% was applied to the transparency of the 3D object: bubble. The alpha value of this object’s colour (albedo) varied gradually from 0 to 50% depending on the moment of the experiment, when the questionnaires were displayed, the alpha value changed from 0 to 50% in two seconds time, giving an effect of “visual focalisation on the questionnaire” displayed inside the bubble.

For the muffling effect, the sound volume was decreased of 50% in two seconds. This created a transition effect to the questionnaire scene, giving the illusion of focusing one’s attention on an exercise while the outside sounds (cheering and singing of the supporters) were still present. The two seconds linear variation for the darkening and muffling effects constituted a soft and continuous transition.

For the blurring effect, the “depth of field” specific Unity post-processing tool, was used. The depth of field is a well-known visual effect technique allowing simulating the eye focus on one object. The image generated by the camera was processed through the standard Unity tool for post processing, and the value of depth of field was changed. The focus point was set at 75 centimetres while the bubble was displayed. Given the technical complexity of this manipulation, this effect was not obtained with a linear variation but happened after the darkening and muffling transition (of two seconds). When the bubble was not displayed, this post processing was not applied to the camera.

To sum up, we can divide the technical sequence of the application into three phases:

- The experience phase: the sphere is transparent (alpha at 0%), the sounds are played at a normal volume (volume at 100%) and the “field of depth” tool is deactivated.
- The transition phase: for two seconds, the alpha value of the colour will be modified linearly from the starting value (0%) to the end value (50%), the same process is applied to the sound volume, the depth of field is activated at the end of these two seconds.
- The concentration phase: the sphere becomes darker (alpha at 50%), the sound volume becomes lower (volume at 50%) and the “depth of field” is activated (blurring after 1.5 metres).

**Visual analogic scales and grid**

Participants had to touch the cursor at least one time to be able to step to the next question. On their right side, there was a floating ball with the word “suivant” (“next” in French). On their left side, there was the same ball with the word “retour” (“back” in French). Participants were thus autonomous and able to go back if they had made a mistake. To activate the blue spheres, participants had to place their virtual hand in contact with it for two seconds, the ball would then change color and the next or previous question would appear in front of them. To avoid the absence of answer from the participants, they had to touch the cursor at least one time, for the button “next” to be active.

To move the cursor, an invisible sphere (of fifteen centimetres diameter) was placed around the participant’s hand. When intersecting the cursor, a collision would be triggered, allowing the displacement of the cursor from one position on the bar to the other. When the participant reached the desired position on the bar, he would remove his hand from the yellow sphere (cursor). The position of the cursor was recorded and saved as its corresponding value between 0 and 100 (the black stripe placed at the right indicated a value of 0, the black stripe placed in the middle indicated a value of 50, and the black stripe placed to the left indicated a value of 100).
