# Supplementary material for: Inducing and measuring identity levels in professional rugby players during immersion in an interactive virtual environment
Source: PLoS One. 2026 Oct 7;21(10):e0359964. doi: 10.1371/journal.pone.0359964 (PMC13645085; doi:10.1371/journal.pone.0359964)
Supplement: Supplementary file 2 [file pone.0359964.s004.docx]

**Supporting information 4. LME and Simple Linear Models Assessing Covariables Influence.**

| **Predictors** | ***β*** | **SE** | ***t*** | ***p*** |
| --- | --- | --- | --- | --- |
| **Social identity** |  |  |  |  |
| **Intercept** | 66.404 | 6.061 | 10.957 | < 0.001 |
| **Time** | 8.539 | 5.865 | 1.456 | 0.149 |
| **Rugby level** | -0.608 | 8.051 | -0.076 | 0.940 |
| **RV experience** | 0.848 | 3.143 | 0.270 | 0.789 |
| **Rugby level × Time** | -11.142 | 7.791 | -1.430 | 0.156 |
| **RV experience × Time** | 2.451 | 3.042 | 0.806 | 0.422 |
| **Personal identity** |  |  |  |  |
| **Intercept** | 45.073 | 6.817 | 6.612 | < 0.001 |
| **Time** | -2.846 | 6.548 | -0.435 | 0.665 |
| **Rugby level** | 6.695 | 9.055 | 0.739 | 0.463 |
| **RV experience** | -0.138 | 3.535 | -0.039 | 0.969 |
| **Rugby level × Time** | 5.109 | 8.698 | 0.587 | 0.558 |
| **RV experience × Time** | -2.343 | 3.396 | -0.690 | 0.492 |
| **Identity dominance** |  |  |  |  |
| **Intercept** | 66.471 | 6.600 | 10.071 | < 0.001 |
| **Time** | 3.800 | 6.681 | 0.569 | 0.571 |
| **Rugby level** | -9.942 | 8.768 | -1.134 | 0.262 |
| **RV experience** | 1.067 | 3.423 | 0.312 | 0.756 |
| **Rugby level × Time** | -7.462 | 8.875 | -0.841 | 0.403 |
| **RV experience × Time** | 3.892 | 3.465 | 1.123 | 0.264 |
| **Valence** |  |  |  |  |
| **Intercept** | 81.195 | 3.861 | 21.031 | < 0.001 |
| **Time** | 4.427 | 3.862 | 1.146 | 0.254 |
| **Rugby level** | -9.199 | 5.129 | -1.794 | 0.079 |
| **RV experience** | -1.690 | 2.002 | -0.844 | 0.403 |
| **Rugby level × Time** | 1.427 | 5.130 | 0.278 | 0.782 |
| **RV experience × Time** | 0.941 | 2.003 | 0.470 | 0.640 |
| **Arousal** |  |  |  |  |
| **Intercept** | 65.557 | 5.586 | 11.737 | < 0.001 |
| **Time** | 9.410 | 4.478 | 23101 | 0.038 |
| **Rugby level** | -5.485 | 7.420 | -0.739 | 0.464 |
| **RV experience** | 2.082 | 2.897 | 0.719 | 0.476 |
| **Rugby level × Time** | 2.497 | 5.947 | 0.420 | 0.676 |
| **RV experience × Time** | -0.897 | 2.322 | -0.386 | 0.700 |
| **Body ownership** |  |  |  |  |
| **Intercept** | 63.616 | 6.092 | 10.443 | < 0.001 |
| **Time** | 6.350 | 4.873 | 1.303 | 0.196 |
| **Rugby level** | -18.831 | 8.093 | -2.327 | 0.025 |
| **RV experience** | 3.696 | 3.159 | 1.170 | 0.249 |
| **Rugby level × Time** | -1.782 | 6.474 | -0.275 | 0.784 |
| **RV experience × Time** | -0.865 | 2.528 | -0.342 | 0.733 |
| **Place illusion** |  |  |  |  |
| **Intercept** | 73.635 | 4.432 | 16.614 | < 0.001 |
| **Time** | 6.199 | 3.412 | 1.817 | 0.072 |
| **Rugby level** | -12.610 | 5.888 | -2.142 | 0.038 |
| **RV experience** | 1.0699 | 2.299 | 0.465 | 0.644 |
| **Rugby level × Time** | -2.066 | 4.532 | -0.461 | 0.646 |
| **RV experience × Time** | 0.990 | 1.769 | 0.559 | 0.577 |
| **Plausibility Illusion** |  |  |  |  |
| **Intercept** | 65.963 | 4.775 | 13.809 | < 0.001 |
| **Rugby level** | -18.765 | 6.343 | -2.958 | 0.004 |
| **RV experience** | 5.046 | 2.476 | 2.038 | 0.046 |
| **Co-presence** |  |  |  |  |
| **Intercept** | 70.309 | 5.057 | 13.904 | < 0.001 |
| **Rugby level** | -8.434 | 6.717 | -1.255 | 0.214 |
| **RV experience** | 1.760 | 2.623 | 0.671 | 0.505 |
